# Esterase D stabilizes FKBP25 to suppress mTORC1

**DOI:** 10.1186/s11658-021-00297-2

**Published:** 2021-12-07

**Authors:** Yuejun Yang, Xinpeng Chen, Wen Yao, Xiaoling Cui, Na Li, ZhaoMin Lin, Baoxiang Zhao, Junying Miao

**Affiliations:** 1 Shandong Provincial Key Laboratory of Animal Cells and Developmental Biology, School of Life Science, Shandong University, Qingdao, 266237 People’s Republic of China; 2 Hubei Key Laboratory of Edible Wild Plants Conservation & Utilization, Hubei Engineering Research Center of Typical Wild Vegetable Breeding and Comprehensive Utilization Technology, Hubei Normal University, Huangshi, 435002 People’s Republic of China; 3 Institute of Medical Science, The Second Hospital of Shandong University, Jinan, 250033 People’s Republic of China; 4 Institute of Organic Chemistry, School of Chemistry and Chemical Engineering, Shandong University, Jinan, 250100 People’s Republic of China

**Keywords:** Esterase D, FKBP25, mTORC1, Ubiquitination, Autophagy

## Abstract

**Background:**

Esterase D (ESD) is a nonspecific esterase that detoxifies formaldehyde. Many reports have stated that ESD activity is associated with a variety of physiological and pathological processes. However, the detailed signaling pathway of ESD remains poorly understood.

**Methods:**

Considering the advantages of the small chemical molecule, our recent work demonstrated that 4-chloro-2-(5-phenyl-1-(pyridin-2-yl)-4,5-dihydro-1*H*-pyrazol-3-yl) phenol (FPD5) activates ESD, and will be a good tool for studying ESD further. Firstly, we determined the interaction between ESD and FK506 binding protein 25 (FKBP25) by yeast two-hybrid assay and co-immunoprecipitation (CO-IP) and analyzed the phosphorylation levels of mTORC1, P70S6K and 4EBP1 by western blot. Furthermore, we used the sulforhodamine B (SRB) and chick chorioallantoic membrane (CAM) assay to analyze cell viability in vitro and in vivo after treatment with ESD activator FPD5*.*

**Results:**

We screened FKBP25 as a candidate protein to interact with ESD by yeast two-hybrid assay. Then we verified the interaction between ESD and endogenous FKBP25 or ectopically expressed GFP-FKBP25 by CO-IP. Moreover, the N-terminus (1–90 aa) domain of FKBP25 served as the crucial element for their interaction. More importantly, ESD reduced the K48-linked poly-ubiquitin chains of FKBP25 and thus stabilized cytoplasmic FKBP25. ESD also promoted FKBP25 to bind more mTORC1, suppressing the activity of mTORC1. In addition, ESD suppressed tumor cell growth in vitro and in vivo through autophagy.

**Conclusions:**

These findings provide novel evidence for elucidating the molecular mechanism of ESD and ubiquitination of FKBP25 to regulate autophagy and cancer cell growth. The ESD/FKBP25/mTORC1 signaling pathway is involved in inhibiting tumor cell growth via regulating autophagy.

**Supplementary Information:**

The online version contains supplementary material available at 10.1186/s11658-021-00297-2.

## Introduction

Autophagy is an important cellular process that generally protects cells and organisms under stressful conditions such as nutrient deprivation. Autophagy is a self-protection program in normal cells, which protects organelle function, breaks down redundant proteins, and maintains basal metabolism in starvation. The role of autophagy in cancer is complicated, depending on biological factors, such as the tumor type and signaling pathway [1, 2]. Autophagy suppresses tumorigenesis in some contexts, whereas it facilitates cancer metabolism and growth in other contexts [3]. Recent studies have reported that autophagy has potential anti-tumor properties [4, 5], but the molecular mechanisms regulating tumor growth still remain to be understood.

Esterase D (ESD) is a nonspecific esterase that detoxifies formaldehyde, expressed in cytoplasmic vesicles of all organisms. Many reports have stated that ESD activity is associated with a variety of physiological and pathological processes. The human ESD gene was cloned in the chromosome 13q14 region in 1986 and suggested as a genetic marker of retinoblastoma [6]. To date, increasing reports have focused on ESD activity and its associated physiological and pathological processes including lung adenocarcinoma, acute myeloid leukemia and virus immune response [7–9]. However, the pathogenic mechanisms of ESD remain poorly understood. Small molecules with the characteristics of cell permeability and selectivity can rapidly and reversibly modulate protein functions [10]. Considering the advantages of the small chemical molecule, our recent work has demonstrated that 4-chloro-2-(5-phenyl-1-(pyridin-2-yl)-4,5-dihydro-1*H*-pyrazol-3-yl) phenol (FPD5) activates ESD and promotes autophagy [11, 12], which will be a good tool for further study of ESD.

FKBP25, high affinity for FK506 or rapamycin, belongs to the immunophilin family with peptidyl-prolyl cis–trans isomerase (PPIase) activity [13, 14]. FKBP25 is composed of a conserved FK506 binding domain (FKBD) at its C-terminus and a unique multi-functional hydrophilic helix-loop-helix (HLH) motif (1–90 aa) at its N-terminal region [15]. Localized primarily in the nucleus, FKBP25 is associated with many important translational factors including transcription factor p53 to participate in gene expression [15–17]. Moreover, FKBP25 is also associated with diverse proteins that form a large intracellular complex such as nucleosome and spliceosome, dsDNA and dsRNA [18–20]. Moreover, some research has revealed that FKBP25 also played various roles in the cytoplasm. FKBP25 interacts with microtubules (MTs) directly to promote their polymerization and stabilize the MT network [21]. Also, rapamycin induces the interaction of FKBP25 with the FK506-rapamycin binding (FRB) domain in living cells [22]. It is still necessary to investigate the detailed mechanisms between FKBP25 and mTORC1. Furthermore, the post-translational modifications and cytoplasmic functions of FKBP25 are still unknown.

Here, we aimed to further investigate the relationship between ESD and FKBP25 in autophagy and their roles in cancer development with the ESD activator FPD5. We found that activated ESD promoted the interaction between ESD and FKBP25, thus enabling stability of cytoplasmic FKBP25. Furthermore, ESD promoted FKBP25 to bind more mTORC1, inhibiting mTORC1 activity to suppress the growth of tumor cells via autophagy, thereby providing new evidence for the novel mechanisms of ubiquitination FKBP25 in regulating autophagy and cancer cell growth.

## Materials and methods

### Antibodies and materials

The small chemical molecule FPD5 was synthesized in the laboratory of Professor Baoxiang Zhao (Shandong University, Jinan). The synthetic protocols were described in detail previously (ref. [23]).

Antibodies for ESD (sc-134333), GFP (sc-8334), ACTB (sc-47778), GAPDH (sc-47724), goat anti-mouse horseradish peroxidase (HRP)-conjugated IgG (GP016129) and goat anti-rabbit HRP-IgG (sc-2004) were purchased from Santa Cruz Biotechnology (Santa Cruz, CA). Mouse anti RFP-Tag mAb (AE020) was from ABclonal. Antibodies for SQSTM1 (610833) were from BD Biosciences. Lipofectamine 2000 transfection reagent (11668-027) and acridine orange (AO, A3568) were from Invitrogen. Normal mouse IgG (sc-2025) and normal rabbit IgG (sc-2027), controls for immunofluorescence and immunoprecipitation assays, were from Santa Cruz Biotechnology. Secondary antibodies for immunofluorescence were goat anti-mouse IgG Alexa Fluor-488 (Invitrogen, A1101) and goat anti-rabbit IgG (Invitrogen, A21070). The antibodies ESD (ab133631) and FKBP25 (ab16654) were purchased from Abcam and mTORC1 (20657-1-AP) was from Proteintech. The antibodies p-mTORC1 (2971s), 4EBP1 (9452s), p-4EBP1 (9459S), P70S6K (9205s), and p-P70S6K (9202s) were from Cell Signaling Technology. SiRNA against ESD or FKBP25 was designed and synthesized by GenePharma (Shanghai). Scramble siRNA was used as a control (Santa Cruz Biotechnology, sc-37007).

### Sulforhodamine B (SRB) assay

Cells were seeded in 96-well plates at 8 × 10^4^ cells/cm^2^ and treated with FPD5 at the indicated concentrations for specified times. Cell viability was measured by SRB assay (Sigma-Aldrich). Briefly, the medium was removed, and cells were fixed by adding 100 μL of cold 10% trichloroacetic acid (Sangon Biotech, Shanghai) and incubated for 1 h at 4 °C. The supernatant was discarded, and plates were washed 5 times with deionized water and dried, then 50 μL of 0.4% (W/V) SRB solution in 1% acetic acid was added to each well for shaking for 5 min on a microtiter plate shaker. Plates were washed 5 times with 1% acetic acid, then dried, and 100 μL of 10 mM unbuffered Tris-base (pH 10.5) was added to dissolve the dye; plates were shaken for 5 min on a microtiter plate shaker and the absorbance was measured at 540 nm wavelength by use of a Spec-traMAX 190 microplate spectrophotometer (GMI Co., USA).

### Plasmid constructs

The coding region of human ESD cDNAs was subcloned into the pEGFP-N1 expression vector with EcoRI and KpnI sites. Mutation changing Lys at positions 213 to Ala was subcloned into the pEGFP-N1 expression vector. All constructs were confirmed by DNA sequencing. Plasmids were designated as pEGFP-N1-*ESD* and pEGFP-N1-*ESD-K213A*, respectively. HEK293T cells were seeded into 6-cm dishes for 24 h. With cells at 70% to 80% confluence, 6 μg of DNA was transfected using Lipofectamine 2000 reagent (Invitrogen, 11668-019).

The plasmids pEGFP-C2 and Myc-C2 were a gift from Professor Zhigang Xu (School of Life Science, Shandong University). The FKBP25 cDNA (GenBank, NM_002013.3) was cloned from total RNA of the A549 cell line and inserted into the plasmids pEGFP-C2 and Myc-C2, then sequenced correctly. The plasmids pEGFP-*FKBP25* and Myc-*FKBP25* were transfected into the HEK293T cell line. Finally, the antibodies GFP and Myc were used to detect the overexpression of fused proteins GFP-FKBP25 and Myc-FKBP25 by immunoblotting.

### Mass spectrometry

FKBP25 in HEK293T cell lysate was collected by FKBP25 antibody and performed by agarose gel electrophoresis. We excised the gel bands of 32 kDa, which were ESD, and dissolved the gel with 100 mM ammonia carbonate. The gel was resolved with 10 mM DTT and 55 mM iodoacetamide, after de-staining. Trypsin (Sigma, T6567, USA) digested the sample at 37 °C overnight. The peptides were extracted twice with 0.1% trifluoroacetic acid (TFA) in 50% acetonitrile aqueous solution for 20 min. Centrifugal reduced the volume of extractions in a speed vac. These peptides were concentrated. Then peptides were purified by ZipTip pipette Tips C18 (Millipore, USA) and dissolved in 0.1% TFA. The LTQ Orbitrap mass spectrometer was operated in the data-dependent acquisition mode using the Xcalibur 3.0 software and there is a single full-scan mass spectrum in the Orbitrap (400–1800 m/z, 30,000 resolution) followed by 20 data-dependent MS/MS scans in the ion trap at 35% normalized collision energy. The MS/MS spectra from each LC–MS/MS run were searched against the selected database using an in-house Mascot or Proteome Discovery searching algorithm.

### Cell staining for immunofluorescence microscopy

Cells were fixed with 4% paraformaldehyde and blocked with normal goat serum (1:30) at room temperature, incubated with primary antibodies (1:200) overnight at 4 °C, then washed with phosphate buffered saline (PBS) three times and incubated with secondary antibody (1:200) for 1 h at 37 °C in the dark. Nuclei were counterstained with DAPI. The fluorescence was captured by an LSM700 (Zeiss, Germany) at the indicated excitation wavelength. The software ZEN 2010 was used to analyze fluorescence intensity in at least 10 regions for each labeling condition, with representative results shown.

### Western blot and immunoprecipitation (IP)

Cell total proteins were extracted with IP buffer (Beyotime, P0013) and collected after centrifuging at 4 °C. Protein concentration was assessed by the Bradford assay and 20–50 μg of protein was separated by SDS-PAGE and then transferred to PVDF (Millipore), which was incubated with primary antibodies at 4 °C overnight. Secondary antibodies were goat antibody to rabbit or anti-mouse IgGs conjugated to horseradish peroxidase (HRP), detected by an enhanced chemiluminescence detection kit (Thermo Fisher, 34080). Infrared secondary antibodies were imaged.

**Fig. 1:**
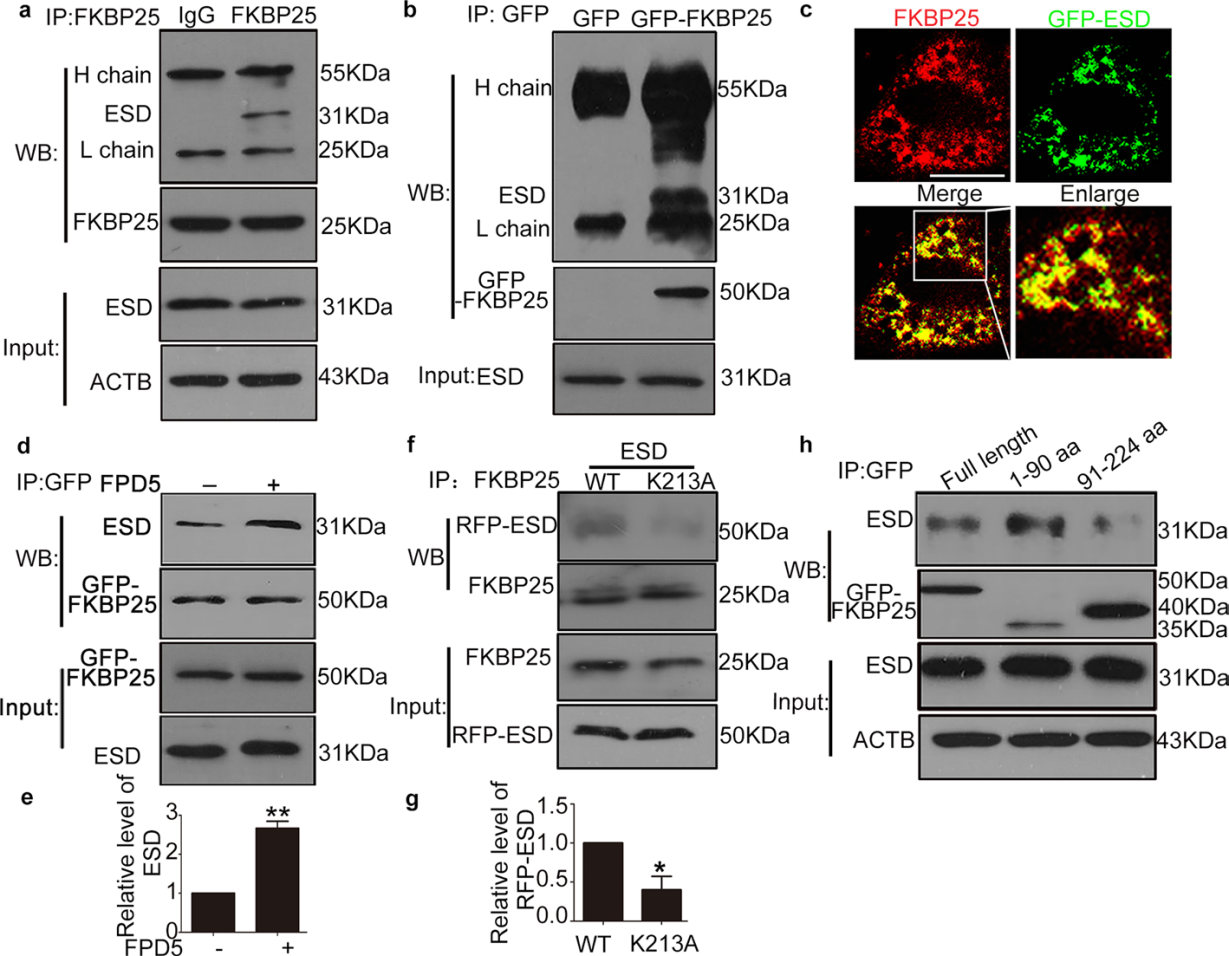
ESD interacted with N-terminus domain of FKBP25 (1–90 aa). **a**, **b** Co-immunoprecipitation (Co-IP) of ESD with the FKBP25 protein or ectopically expressed GFP-FKBP25 from HEK293T cells. **c** Double immunofluorescence staining of GFP-ESD (green) and FKBP25 (red) showed agglomeration and co-localization in HeLa cells. Overlay of the GFP-ESD and FKBP25 with yellow indicates co-localization. Scale bar: 25 μm. **d**, **e** A549 cell line was transfected with plasmid pEGFP-FKBP25 for 24 h and then exposed to FPD5 (5 μM) for 24 h. Co-IP showed that ESD could bind more FKBP25 induced by FPD5. **f**, **g** HEK293T cell line was transfected with plasmids pCAG-ESD-WT and pCAG-*ESD-K213A* for 48 h. Then western blot analysis of co-immunoprecipitation of RFP-ESD with anti-FKBP25 antibodies and quantification of RFP-ESD level. **h** HEK293T cell line was transfected with plasmids FKBP25-WT and the deletion mutants of FKBP25 for 24 h respectively. Then the levels of GFP-FKBP25 and ESD were analyzed after Co-IP of ESD with anti-GFP antibodies. Data are mean ± SEM. *p < 0.05, **p < 0.01, n = 3

For immunoprecipitation, lysates (1 mg of protein) of cells transfected with different variants of GFP-FKBP25, GFP-FKBP25-1-90aa, GFP-FKBP25-91-224aa, RFP-ESD, and/or RFP-ESD-K213A were incubated with 1 μg of primary antibody overnight at 4 °C [rabbit anti-ESD (ab133631) and rabbit anti-FKBP25 (ab16654) from Abcam in Fig. 1a, mouse anti-ESD (sc-134333) and mouse anti-GFP (sc-8334) from Santa Cruz Biotechnology in Fig. 1b, rabbit anti-ESD (ab133631) and mouse anti- GFP (sc-8334) in Fig. 1d, h and mouse anti-RFP (sc-8334) and rabbit anti-ESD (ab133631) in Fig. 1f], containing 20 μL of protein A/G Sepharose beads (Beyotime). Immunoprecipitates were then eluted with IP buffer, boiled with 2×SDS loading buffer and subjected to western blot.

### Cell culture

HeLa and HEK293 cells were cultivated with Dulbecco’s modified Eagle’s medium (DMEM, Gibco, 12800-058) which contained 10% fetal bovine serum (FBS; v/v) (Hyclone, SV30087.02). RPMI medium 1640 (Gibco, 3180-022) with 10% FBS was used for A549, H322 and human umbilical vein endothelial cells (HUVEC) cell line growth. All cells were incubated at 37 °C in a humidified incubator with 5% CO_2_.

### Establishment of ESD knockout cell line by CRISPR/Cas9

The Cas9/sgRNA (pGK1.1-*ESD*) plasmid vector was constructed by Shandong Shengqiangde Biotechnology Co., Ltd. HEK293T cells were plated in 6-well plates and infected with 4 μg of pGK1.1-*ESD* plasmid using Lipofectamine 2000 (Invitrogen, 1803709). After 24 h, transfected positive cells were screened by puromycin (3 μg/mL) for another 48 h. Then, the positive cells were diluted to obtain a single cell and then seeded on a 96-well plate. Single cell was grown into a single clone after 1 month. Finally, we measured ESD expression through gene sequencing and western blot.

### Yeast two-hybrid assay

Briefly, the vector pGBKT7 (Clontech, 630489) of full-length human *ESD* cDNA (GenBank, NM_001984.1) and the pACT2 vector (Clontech, 638822) of a human liver cDNA library were constructed and transfected into the yeast strain AH109 (Clontech, 630444). 5 × 10^6^ transfected yeasts were selected in the presence of HIS3 (2.5 mM 3-amino-1,2,4-triazole) and the positive colonies were verified by reporter genes ADE2 and lacZ. Then inserted cDNA of the positive colonies were sequenced.

### RNA interference (RNAi)

To knock down the expression of ESD or FKBP25, RNA interference was performed as follows. Briefly, the A549 cell line was transfected with ESD or FKBP25 siRNA (40 nM) for 24 h using Lipofectamine 2000 (Invitrogen, 1803709) according to instructions, then gene knockdown efficiency was evaluated by western blot. The siRNA ESD and siRNA FKBP25 were synthesized and purchased from GenePharma (Shanghai, China). SiRNA ESD sequence (5ʹ–3ʹ): GCUACCCACCUUGUGAAAUTT, AUUUCACAAGGUGGGUAGCTT. SiRNA FKBP25 sequence (5ʹ–3ʹ): CCACUUGGUUACAGCCUAUTT, AUAGGCUGUAACCAAGUGGTT.

### In vivo tumor assay of chick embryo chorioallantoic membrane (CAM)

Eight-days-old fertilized chicken eggs were seeded with eight million A549 lung cancer cells in 20 μL of medium on the silicone ring at 37 °C with 60% relative humidity. After 2 days, FDP5 at different doses (0 μM, 10 μM, 50 μM and 100 μM) was added into eggs every 2 days for 3 times. Then the CAM and tumors were collected and photographed. Tumor size matching was based on tumor volume calculation: length × width × width × 0.5. All animal studies were approved by the Animal Care and Use Committees of Shandong University of School of Life Science (Approval No. SYDWLL-2017-04 from March 1 2017 to December 31 2021) and performed in adherence with the Basel Declaration and the institutional guidelines for the care and use of animals.

### Angiogenesis assay of CAM in vivo

Fertilized chicken eggs of embryonic day 9 were treated with FPD5 (50 μM) or DMSO every 2 days. After 6 days, angiogenesis of the treated CAM was sampled, photographed and analyzed with Image-Pro Plus [24].

### Quantitative analysis and statistical analysis

The quantification of western blot and MAP1LC3B puncta was analyzed by software Image J 1.44P. Data are expressed as mean ± SEM. SPSS 11.5 (SPSS Inc., Chicago, IL) was used for analysis. Data were analyzed by one-way ANOVA (followed by the Scheffé F test for post-hoc analysis). P < 0.05 was considered statistically significant.

## Results

### ESD interacted with N-terminus domain of FKBP25 (1–90 aa)

Reduced expression or activity of ESD is closely associated with many diseases [7–9]. However, the detailed mechanism and signal pathway that ESD is involved in remain poorly understood. Firstly, we performed yeast two-hybrid assay to find candidate proteins of this signal pathway. *ESD* cDNA was cloned into plasmid pGBKT7 and then transfected into yeast as bait protein to capture prey proteins with high throughput. All positive clones were picked up, and then their DNA was extracted and sequenced. FKBP25 was identified as a candidate factor (Additional file 1: Fig. S1a). Then we verified the interaction between ESD and endogenous FKBP25 (Fig. 1a) or ectopically expressed GFP-FKBP25 (Fig. 1b) by co-immunoprecipitation. Furthermore, we successfully observed the co-localization of GFP-ESD and FKBP25 with immunofluorescence in HeLa cells (Fig. 1c). In addition, the unique peptide of ESD (SGYHQSASEHGL) was also detected by MS in the sample that was enriched with FKBP25 antibody in the whole cell lysate (Additional file 1: Fig. S1b). Therefore, ESD indeed interacted with FKBP25.

FPD5, an effective ESD activator, could promote the interaction between ESD and FKBP25 (Fig. 1d, e). We further examined whether the activity of ESD affected their interaction. Lys213 of ESD is reported to be important for ESD activity [12]. The data showed that the mutant ESD-K213A significantly reduced their interaction compared to wild type ESD (Fig. 1f, g). Therefore, ESD activity significantly altered their interaction. FKBP25 shares a common domain of PPIase family at the C terminus (90–224 aa) and a DNA binding domain at the N terminus (1–90 aa) [17, 22]. We wondered which domain of FKBP25 was responsible for their interaction. Two individual plasmids expressing the FKBP25 mutant truncations with the GFP tag were constructed and transfected into the HEK293T cell line. The results showed that the N-terminus (1–90 aa) domain of FKBP25 served as the crucial element for their interaction (Fig. 1h).

### ESD suppressed mTORC1 activity

**Fig. 2:**
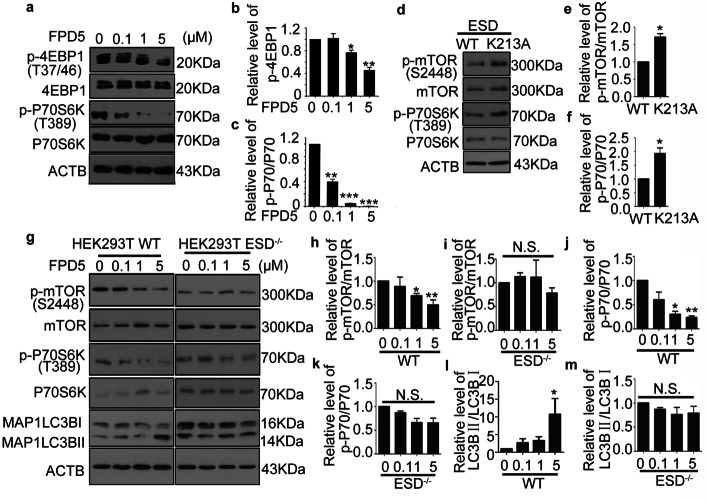
ESD suppressed mTORC1 activity. **a**–**c** Western blot analysis of 4EBP1, phosphorylated 4EBP1 (p-4EBP1, Thr37/46), P70S6K, and phosphorylated P70S6K (p-P70S6K, Thr389) level in A549 cells treated with FPD5 at 0–5 μM for 24 h. **d**–**f** HEK293T cell line was transfected with plasmids pCAG-ESD-WT and pCAG-*ESD*-*K213A* for 48 h followed by western blot analysis of the level of p-mTORC1 and p-P70S6K. **g**–**m** Western blot analysis of mTORC1, phosphorylated mTORC1, P70S6K, phosphorylated P70S6K (p-P70S6K, Thr389) and MAP1LC3BII level in HEK293T-WT and HEK293T-ESD^−/−^ treated with FPD5 at 0.1–5 μM for 24 h. Data are mean ± SEM. *p < 0.05, **p < 0.01, ***p < 0.001; N.S., not significant, n = 3

It has been reported that FPD5 activates ESD activity and promotes autophagy flux [12]. Over the past 20 years of research, mammalian target of rapamycin (mTOR) has been shown to have a crucial role in negatively regulating autophagy [25]. Therefore, to determine whether ESD regulated autophagy via mTORC1, the two substrates of mTORC1, eukaryotic translation initiation factor 4E-binding protein 1 (4EBP1) and ribosomal protein S6 kinase beta-1 (P70S6K), were investigated after treatment with different doses of FPD5 for 24 h. The phosphorylation levels of mTORC1 and its two substrates (P70S6K and 4EBP1) were obviously decreased in an FPD5 dose-dependent way (Fig. 2a–c), thus indicating that mTORC1 activity was suppressed. Next, we observed that FPD5 decreased the phosphorylation levels of mTORC1 and its substrates in A549 cells transfected with scramble siRNA, but not in cells transfected with ESD siRNA (Additional file 2: Fig. S2a–e). In addition, we observed that ESD-K213A did not suppress mTORC1 activity compared to wild type ESD (Fig. 2d–f). These data suggested that mTORC1 activity was restrained by activated ESD. To further clarify that ESD could promote autophagy via suppressing mTORC1 activity, 3BDO, an effective activator of mTORC1 [26], was added into the A549 cell line in the presence of FPD5. Interestingly, we found that after the treatment with 3BDO, FPD5 did not decrease the phosphorylation level of 4EBP1 any more (Additional file 2: Fig. S2f, g).

To further investigate the relationship between ESD and mTORC1, the ESD knockout HEK293T cell line was constructed with CRISPR/Cas9 gene-editing technology [27]. Similarly, the phosphorylation levels of mTORC1 and P70S6K were gradually decreased in an FPD5 dose-dependent way in the HEK293T cell line. However, FPD5 did not alter the phosphorylation levels of mTORC1 and P70S6K of the HEK293T ESD-knockout cell line (Fig. 2g–k). Consistently, the level of MAP1LC3BII also significantly increased after treatment with FPD5 at 5 μM for 24 h, but not in the ESD-knockout cell line (Fig. 2g, l, m). These data suggested that ESD might suppress mTORC1 activity to promote autophagy.

### FKBP25 was essential for ESD suppressing mTORC1 activity

**Fig. 3:**
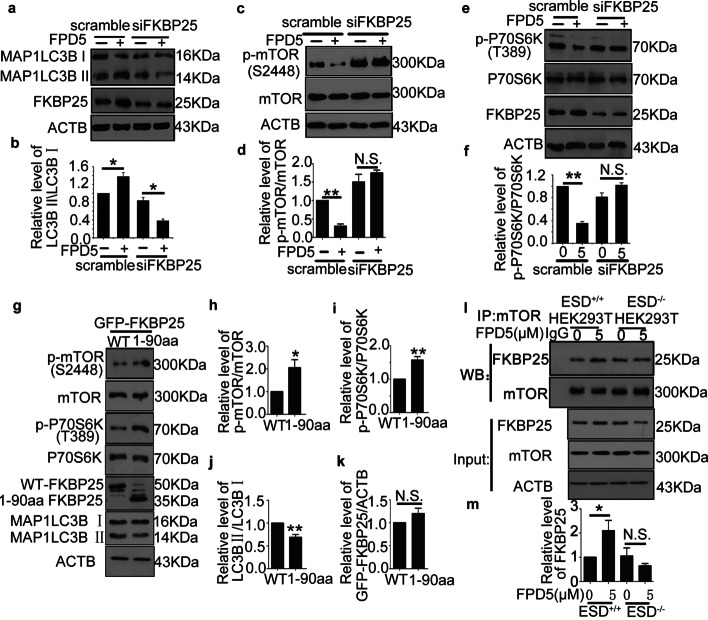
FKBP25 was essential for ESD suppressing mTORC1 activity. Western blot analysis of MAP1LC3BII (**a**, **b**), mTORC1, phosphorylated mTORC1 (p-mTORC1, Ser2448) (**c**, **d**), P70S6K and phosphorylated P70S6K (p-P70S6K, Thr389) level (**e**, **f**) in A549 cells treated with FPD5 at 5 μM for 24 h after transfection with scramble siRNA or specific siRNA for FKBP25 (siFKBP25). **g**–**k** HEK293T cell line was transfected with plasmids FKBP25-WT and the deletion mutants of FKBP25 for 24 h followed by western blot analysis of the levels of p-mTORC1, p-P70S6K, MAP1LC3BII and GFP-FKBP25. **l**, **m** The interaction between FKBP25 and mTORC1 in wild-type HEK293T cells and the ESD-knockout cell line (ESD^−/−^) treated with FPD5 (5 μΜ) for 24 h. Data are mean ± SEM. *p < 0.05, **p < 0.01, N.S., not significant, n = 3

Like FKBP12, FKBP25 is a member of the PPIase family and interacts with FK506-rapamycin binding (FRB) in the presence of rapamycin [22]. However, the effect of FKBP25 on autophagy remains obscure. We noted that MAP1LC3BII was decreased with increased efficiency of FKBP25 siRNA (Additional file 3: Fig. S3a–c), indicating that FKBP25 had a positive role in autophagy**.** Furthermore, knockdown of FKBP25 had no obvious effect on the protein level of ESD (Additional file 3: Fig. S3d–f). We also observed that the level of MAP1LC3II was significantly increased by treatment with FPD5 in A549 cells in the mock group, but knockdown of FKBP25 completely abolished the effect of FPD5 (Fig. 3a, b). Moreover, FPD5 significantly increased the level of MAP1LC3BII after overexpression of FKBP25 (Additional file 3: Fig. S3g, h). We wondered whether FKBP25 participated in FPD5-evoked autophagy. As shown in Fig. 3c–f, FPD5 could significantly decrease the phosphorylation levels of mTORC1 and its substrate P70S6K in the mock group, but this effect of FPD5 was also abolished when FKBP25 was knocked down. The data proved that FKBP25 participated in FPD5-evoked autophagy via mTORC1.

**Fig. 4:**
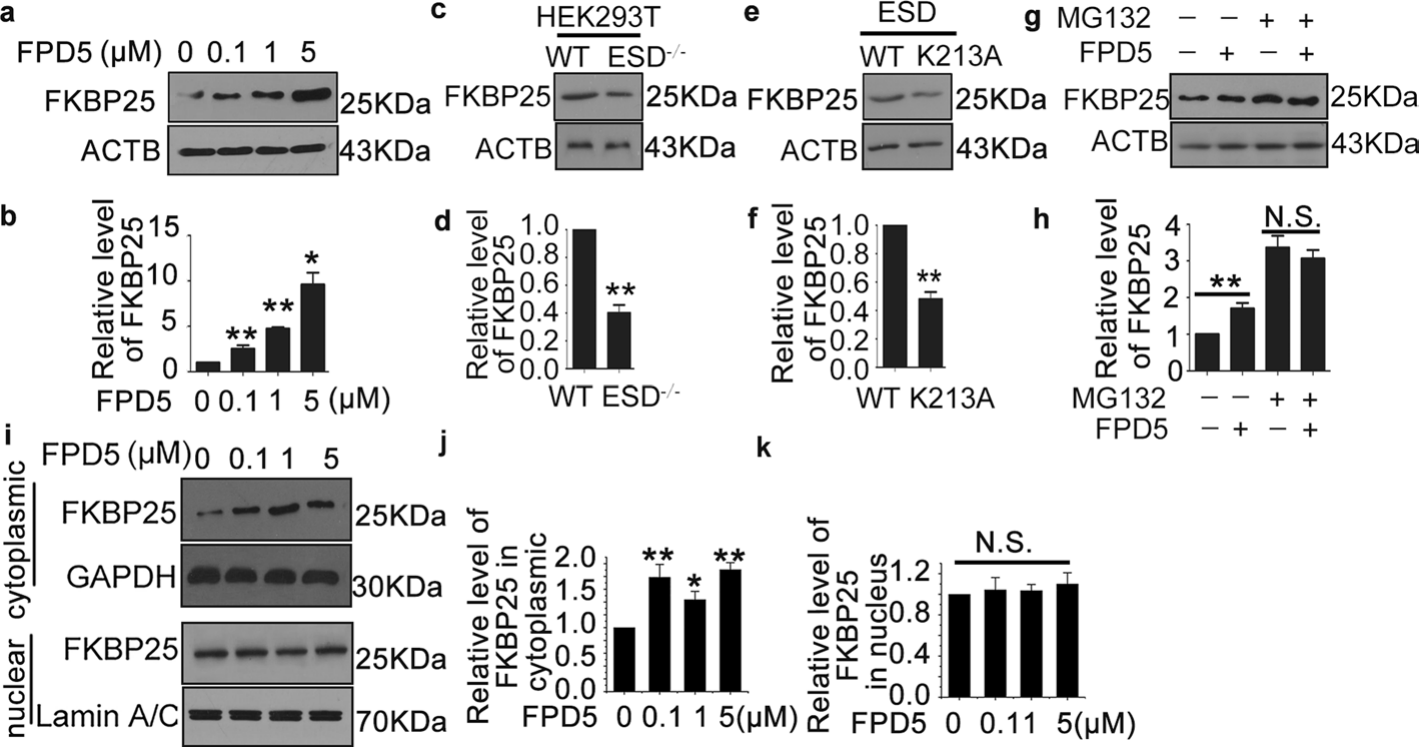
ESD stabilized cytoplasmic FKBP25. **a**, **b** Western blot analysis of FKBP25 and ACTB level in A549 cells treated with FPD5 at 0–5 μM for 24 h. **c**, **d** Western blot analysis of FKBP25 level in HEK293T-WT and HEK293T-ESD^−/−^. **e**, **f** HEK293T cell line was transfected with plasmids pCAG-ESD-WT and pCAG-ESD-K213A for 48 h. Then the level of FKBP25 was analyzed by western blot. **g**, **h** Western blot analysis of FKBP25 and ACTB level in A549 cells treated with FPD5 at 5 μM for 24 h in the presence of proteasome inhibitor MG132. **i**–**k** Western blot analysis of FKBP25, GAPDH and lamin A/C level in cytoplasm and nucleus of A549 cells treated with FPD5 at 0–5 μM for 24 h. Data are mean ± SEM. *p < 0.05, **p < 0.01, N.S., not significant, n = 3

We also transfected the two plasmids expressing the wild type FKBP25 and FKBP25 mutant (1–90 aa) into the HEK293T cell line (Fig. 3g) and observed that the FKBP25 mutant could increase p-mTORC1, p-P70S6K (Fig. 3g–i) and p-4EBP1 (Additional file 3: Fig. S3i, j) and decrease MAP1LC3BII (Fig. 3g, j, k), indicating that the element (90–224 aa) of FKBP25 might suppress the activity of mTORC1, which is consistent with previous finding [15]. Furthermore, we found that activated ESD contributed to the interaction between FKBP25 and mTORC1 in wild-type HEK293T cells treated with FPD5, but not in the ESD-knockout cell line (Fig. 3l, m). These data indicated that ESD promoted the interaction between FKBP25 and mTORC1, which resulted in suppressing mTORC1 activity and promoting autophagy.

### ESD stabilized cytoplasmic FKBP25

Next, we tried to determine why ESD promoted the interaction between FKBP25 and mTORC1. Firstly, we found that the level of total FKBP25 was obviously increased with FPD5 treatment in a concentration-dependent manner (Fig. 4a, b). Unlike the wild-type cells, the level of total FKBP25 was much lower in the ESD-knockout cell line (Fig. 4c, d), indicating that ESD might stabilize FKBP25. To confirm the phenomenon, we also examined the level of FKBP25 after expressing the mutant ESD K213A. The mutant also impaired the stability of FKBP25 (Fig. 4e, f).

Since the ubiquitin–proteasome degradation pathway is closely associated with protein quality control [28], we next observed whether or not the protein level of FKBP25 was changed after treatment with FPD5 in the presence of the proteasome inhibitor MG132 [29]. FPD5 could increase the level of FKBP25 in the absence of MG132 (Fig. 4g, h). However, when the proteasome pathway was blocked to inhibit protein degradation with MG132, FPD5 did not significantly upregulate FKBP25 any more (Fig. 4g, h), indicating that ESD protected FKBP25 from degradation through proteasome. Furthermore, there were various forms of poly-ubiquitin chains, such as Lys11, Lys33, Lys48 and Lys63 linked ubiquitin chain. It was suggested that K48-linked ubiquitin chains were committed to the proteasomal degradation pathway [30, 31]. The data indicated that the level of K48-linked poly-ubiquitin chains also reduced significantly after the treatment with FPD5 (Additional file 4: Fig. S4).

It is reported that FKBP25 is mainly located at the nucleus and binds directly to DNA and some transcription factors [15, 16, 32]. We also examined the fraction of FKBP25 in the cytoplasm or nucleus by western blot. The data showed that FPD5 increased cytoplasmic FKBP25 but not nuclear FKBP25 (Fig. 4i–k). Therefore, ESD enabled cytoplasmic FKBP25 stability and reduced the proteasomal degradation via K48 linked poly-ubiquitination.

### ESD activated by FPD5 inhibited cancer cell growth in vitro and in vivo

**Fig. 5:**
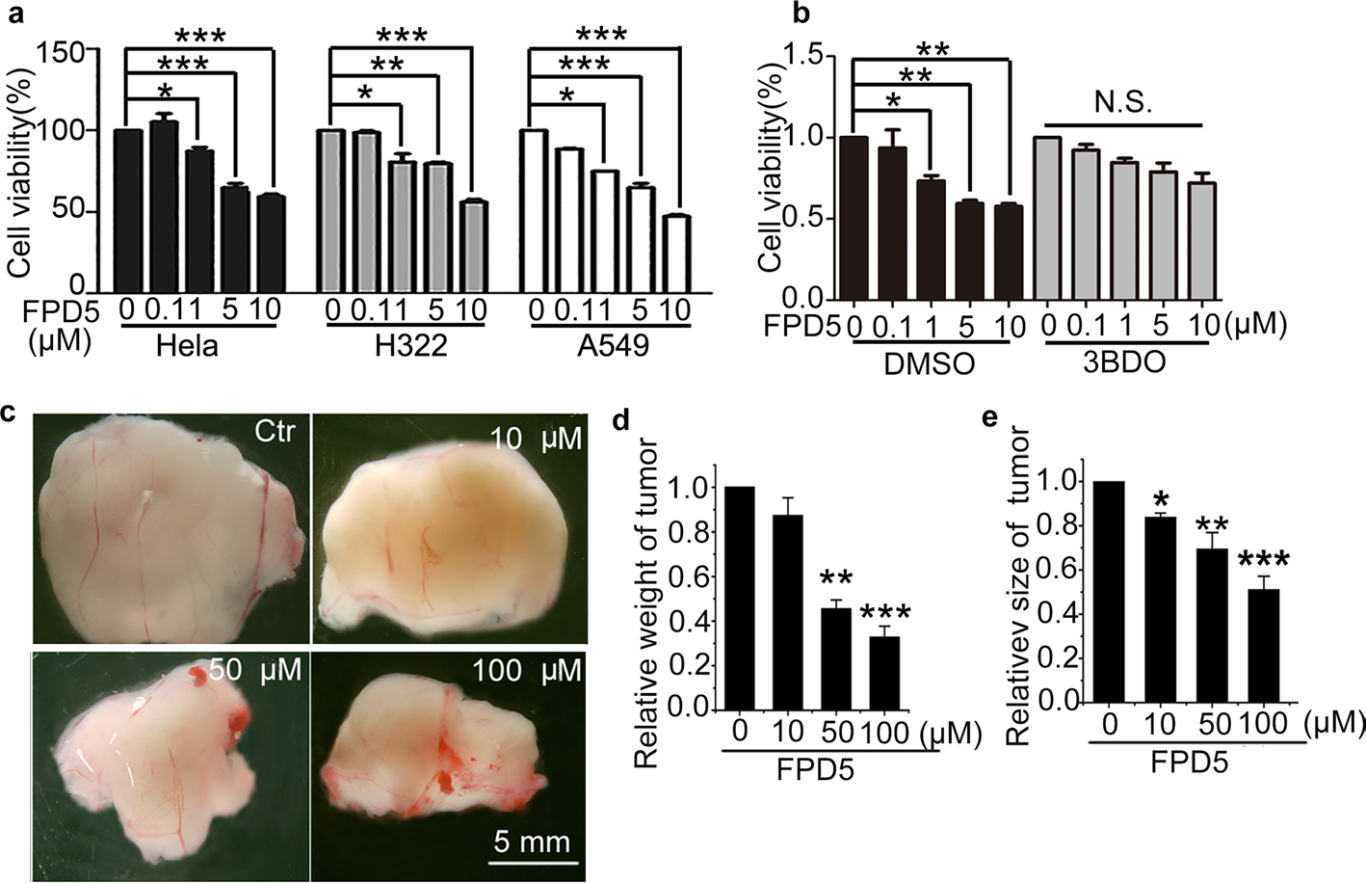
Activated ESD induced by FPD5 inhibited cancer cell growth in vitro and in vivo. **a** FPD5 at 1–10 μM for 24 h decreased A549, H322 and HeLa cell viability. **b** The effect of FPD5 at 0.1–10 μM for 24 h on A549 cell viability in the presence of mTORC1 activator 3BDO. **c**–**e** FPD5 at different doses (10, 50, and 100 μM) reduced the size and weight of the tumor on CAM. Representative images are shown. Scale bar: 5 mm. Data are mean ± SEM. *p < 0.05, **p < 0.01, ***p < 0.001, N.S., not significant, n = 3

Since recent studies have reported that autophagy has potential anti-tumor properties [4, 5], we sought to determine the biological effect of FPD5 on cancer cell growth. FPD5 (i.e., 1, 5, 10 µM) significantly decreased the viability of A549, H322 or HeLa cells at 24 h (Fig. 5a). We found that FPD5 dad no obvious effect on the cleavage level of poly (ADP-ribose) polymerase (PARP) or the level of bax at different doses (i.e., 0.1, 1, 5, and 10 μM) in A549 cells (Additional file 5: Fig. S5a–c). We also examined the effect of FPD5 on normal cells and found that FPD5 did not induce apoptosis in human umbilical vein endothelial cells (HUVEC) (Additional file 5: Fig. S5d–f). To determine whether the inhibitory growth effect of FPD5 was due to necrosis, we performed lactate dehydrogenase (LDH) assays in cells with FPD5 or DMSO (as a control). No noticeable change of LDH activity was found after FPD5 treatment at the tested range of concentrations, suggesting that FPD5 did not induce necrosis in cancer cells (Additional file 5: Fig. S5g). Since the inhibitory growth effect of FPD5 was not due to apoptosis and necrosis, we wondered whether autophagy played a vital role in this effect [33, 34]. 3BDO was reported to be an effective activator of mTORC1 [26]. We found that the viability of A549 cells treated with FPD5 decreased, but 3BDO completely abolished the inhibitory effects of FPD5 (Fig. 5b). It seemed that the compromised cell viability of cancer cells by FPD5 was through autophagy, not necrosis or apoptosis.

In order to simulate the internal environment of living organisms, the chick chorioallantoic membrane (CAM) assay has been widely utilized to study tumor growth, angiogenic and anti-angiogenic molecules [35, 36], since it has an immune-deficient environment and the angiogenesis network [24]. So we performed a CAM assay with FPD5. The results indicated that the transplanted cancer size and weight decreased significantly at FPD5 concentrations of 50 μM or 100 μM (Fig. 5c–e). Most importantly, the angiogenesis of CAM was normal with FPD5 (Additional file 5: Fig. S5h, i). Therefore, ESD activated by FPD5 inhibited cancer cell growth in vitro and in vivo*.*

## Discussion

Autophagy is a double-edged sword in the development of cancer: it clears the damaged organelles and proteins, thus serving as a tumor suppressor, but also can promote the survival of tumor cells by degrading cargoes for energy production [37–39]. In humans, ESD is expressed in most tissues, with greater activity in the liver, kidney, and placenta than other tissues. Although increasing reports have demonstrated that ESD activity is involved in many pathological processes [7, 8, 40], it is still largely unknown how ESD participates in these physiological processes. Recently, we found that the small molecule FPD5, an ESD activator, will be a good tool for studying ESD [12]. Firstly, a new factor, FKBP25, was sought out and its N-terminus was found to interact with ESD. We also revealed that ESD is involved in autophagy via the FKBP25/mTORC1 axis in A549 cells by using FPD5. Moreover, we also found that FPD5-activated ESD could inhibit the growth of cancer cell in vitro and in vivo, suggesting that ESD is a potential anti-tumor drug target. In addition, it could significantly induce autophagy but not cell apoptosis and necrosis at a high dose, indicating that autophagy plays a vital role in suppressing cell viability of cancer cells. Therefore, our results extend the scope of our study, emphasizing the novel role of ESD in controlling autophagy signaling.

The mTORC1 complex plays a central role in autophagy [41]. Our data showed that FKBP25 could directly interact with mTORC1 in vivo without rapamycin to inhibit mTORC1 activity, as an endogenous inhibitor of mTORC1 [22, 42]. Our model predicts that ESD is a crucial factor to mediate the interaction between FKBP25 and mTORC1. Our previous evidence has shown that ESD accumulates in the lysosome [27]. mTORC1 is also known to be recruited to and located on the membrane of the lysosome, which changes when nutrient availability changes [43]. It is possible that ESD recruits FKBP25 to the lysosome localization to inhibit mTORC1. However, this needs to be investigated further. In addition, we found that the 1–90 aa FKBP25 could increase p-mTORC1, p-P70S6K and p-4EBP1 and decrease MAP1LC3BII, indicating that the C-terminus (90–224 aa) of FKBP25 might play a role in regulating the activity of mTORC1. These results are consistent with previous reports that FKBP25 comprises a conserved FK506 binding domain (FKBD) at its C-terminus [15]. It has been reported that cancer cells depend on PI3K-Akt-mTOR signaling for survival in response to DNA damage [44], indicating that regulating autophagy is a good tool to inhibit tumors. Therefore, ESD and FKBP25 could be good candidate factors to regulate mTORC1 as a tumor suppressor in cancer cells.

Moreover, we clarified that activated ESD could bind to more FKBP25 and reduce the K48 linked poly-ubiquitination of FKBP25 that is associated closely with the ubiquitin–proteasome degradation pathway, increasing the level of FKBP25 to suppress mTORC1. It also implies that the particular ubiquitin ligase E3 might participate in this process. In a previous study, we also found that ESD interacted with JAB1 [27], which cleaved the ubiquitin-like protein Nedd8 from the Cul1 subunit of SCF ubiquitin ligases to regulate the ubiquitin process [45]. Therefore, we suggested that ESD bound to FKBP25, blocking E3 ligase access to FKBP25. Ubiquitination was found to be involved in many biochemical processes and in a dynamic balance [46]. The binding protein plays an important role in modulating the ubiquitination balance and blocks E3 ligase access to the protein [47]. The binding of ESD might alter the conformation of FKBP25. Therefore, E3 ligase could not recognize its substrate FKBP25. In addition, the interaction of ESD and FKBP25 contributed to understanding the precise molecular mechanism of ESD and its physiological function in signal transduction.

**Fig. 6:**
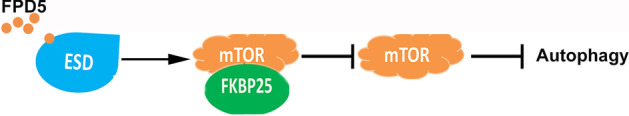
Schematic diagram illustrating the mechanism of autophagy via ESD/FKBP25/mTORC1

In summary, we found the novel relationship between ESD and FKBP25 in autophagy and their roles in cancer development with the ESD activator FPD5. First, we demonstrated that activated ESD induced by FPD5 promoted the interaction between ESD and FKBP25, thus reducing the K48-linked poly-ubiquitin chains of FKBP25, promoting FKBP25 to bind more mTORC1 and suppressing the activity of mTORC1. FKBP25 effectively inhibited tumor cell growth in vitro and in vivo via inhibiting mTORC1 dependent autophagy, thereby providing new evidence for the post-translational modifications of FKBP25 and the molecular mechanism of ESD to regulate autophagy and cancer cell growth (Fig. 6).

## Supplementary Information

**Additional file 1: Figure S1.** FKBP25 interacted with ESD. (**a**) Yeast two-hybrid assays were performed to search for proteins that interacted with ESD. A fusion construct of the GAL4 DNA-binding domain with ESD was constructed as bait for screening a human liver yeast two-hybrid cDNA library, and FKBP25 was identified. (**b**) FKBP25 in HEK293T cell lysate was collected by FKBP25 antibody and was separated by SDS-PAGE. The band corresponding to ESD (SGYHQSASEHGL) was identified by mass spectrometry.

**Additional file 2: Figure S2.** FPD5 participated in regulation of mTORC1 activity via ESD. (**a**–**e**) Western blot analysis of mTORC1, phosphorylated mTOR (p-mTOR, Ser2448), 4EBP1, phosphorylated 4EBP1 (p-4EBP1, Thr37/46), p70S6K and phosphorylated p70S6K (p-p70S6K, Thr389) level in A549 cell treated with FPD5 at 5 μM for 24 h after transfected with scramble siRNA or specific siRNA for ESD (siESD). (**f**, **g**) Western blot analysis of 4EBP1 and phosphorylated 4EBP1 (p-4EBP1, Thr37/46) level in A549 cell treated with FPD5 at 5 μM and 3BDO for 24 h. Data are mean ± SEM. *p < 0.05, **p < 0.01, N.S., not significant, n = 3.

**Additional file 3: Figure S3.** FKBP25 suppressed autophagy. (**a**–**c**) Western blot analysis of MAP1LC3BII and SQSTM1 level in A549 cell after transfected with scramble siRNA or specific siRNA for FKBP25 (siFKBP25). (**d**) Western blotting analysis of ESD and FKBP25 in HEK293T cells transfected with scrambled siRNA (scramble) or siRNA-FKBP25 (siFKBP25) for 24 h. Relative protein levels of FKBP25 and ESD is a ratio to ACTB (**e**–**f**). (**g**, **h**) Western blot analysis of MAP1LC3BII and SQSTM1 level in A549 cell treated with FPD5 at 0–5 μM for 24 h after transfected with myc-FKBP25. (**i**, **j**) HEK293T cell line was transfected with plasmids FKBP25-WT and the 1–90 aa FKBP25 for 24 h respectively. Then Western blot analyzed the levels of p-4EBP1 and 4EBP1. Data are mean ± SEM. *p < 0.05, **p < 0.01, ***p < 0.001, N.S., not significant, n = 3.

**Additional file 4: Figure S4.** ESD reduced the K48-linked polyubiquitination of FKBP25 after treatment with FPD5 at 5 μM for 24 h.

**Additional file 5: Figure S5.** FPD5 did not induce apoptosis and necrosis. (**a**–**c**) FPD5 at 1–10 μM for 24 h decreased A549, **H322** and HeLa cell viability. (**d**–**f**) Western blot analysis of cleavage PARP and Bax level in normal HUVEC cells treated with FPD5 at 0.1–10 μM for 24 h. (**g**) Lactate dehydrogenase (LDH) assay were performed in cancer cells with FPD5 at 10 μM for 24 h. (**h**, **i**) Biomicroscopy and quantification of angiogenesis on gelatin sponge with FPD5 adsorption. Scale bar: 1.5 mm. Data are mean ± SEM. N.S., not significant, n = 3.

## Data Availability

The authors confirm that the data supporting the findings of this study are available within the article and its Additional files.

## Abbreviations

ESD: Esterase D
FKBP25: FK506 binding protein 25
SRB: Sulforhodamine B
CAM: Chick chorioallantoic membrane
CO-IP: Co-immunoprecipitation
mTOR: Mammalian target of rapamycin
4EBP1: 4E-binding protein 1
P70S6K: Ribosomal protein S6 kinase beta-1
LDH: Lactate dehydrogenase
PARP: Poly (ADP-ribose) polymerase
FKBD: FK506 binding domain
HUVEC: Human umbilical vein endothelial cells
