# Supplementary material for: Esterase D stabilizes FKBP25 to suppress mTORC1
Source: Cell Mol Biol Lett. 2021 Dec 7;26:50. doi: 10.1186/s11658-021-00297-2 (PMC8903700; doi:10.1186/s11658-021-00297-2)
Supplement: Supplementary file 1 — Additional file 1: Figure S1. FKBP25 interacted with ESD. (a) Yeast two-hybrid assays were performed to search for proteins that interacted with ESD. A fusion construct of the GAL4 DNA-binding domain with ESD was constructed as bait for screening a human liver yeast two-hybrid cDNA library, and FKBP25 was identified. (b) FKBP25 in HEK293T cell lysate was collected by FKBP25 antibody and was separated by SDS-PAGE. The band corresponding to ESD (SGYHQSASEHGL) was identified by mass spectrometry. [file 11658_2021_297_MOESM1_ESM.docx]

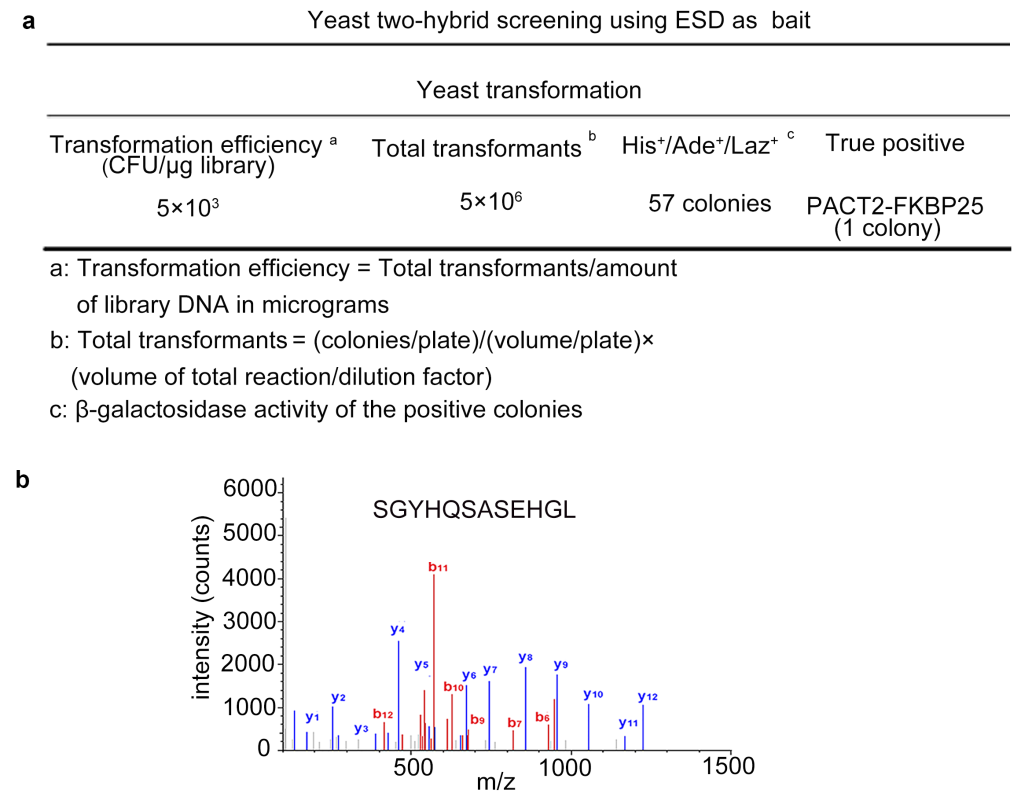


**Additional file 1: Fig. S1.** **FKBP25 interacted with ESD.** (**a**) Yeast two-hybrid assays were performed to search for proteins that interacted with ESD. A fusion construct of the GAL4 DNA-binding domain with ESD was constructed as bait for screening a human liver yeast two-hybrid cDNA library, and FKBP25 was identified. (**b**) FKBP25 in HEK293T cell lysate was collected by FKBP25 antibody and was separated by SDS-PAGE. The band corresponding to ESD (SGYHQSASEHGL) was identified by mass spectrometry.
