# Supplementary material for: Esterase D stabilizes FKBP25 to suppress mTORC1
Source: Cell Mol Biol Lett. 2021 Dec 7;26:50. doi: 10.1186/s11658-021-00297-2 (PMC8903700; doi:10.1186/s11658-021-00297-2)
Supplement: Supplementary file 2 — Additional file 2: Figure S2. FPD5 participated in regulation of mTORC1 activity via ESD. (a–e) Western blot analysis of mTORC1, phosphorylated mTOR (p-mTOR, Ser2448), 4EBP1, phosphorylated 4EBP1 (p-4EBP1, Thr37/46), p70S6K and phosphorylated p70S6K (p-p70S6K, Thr389) level in A549 cell treated with FPD5 at 5 μM for 24 h after transfected with scramble siRNA or specific siRNA for ESD (siESD). (f, g) Western blot analysis of 4EBP1 and phosphorylated 4EBP1 (p-4EBP1, Thr37/46) level in A549 cell treated with FPD5 at 5 μM and 3BDO for 24 h. Data are mean ± SEM. *p < 0.05, **p < 0.01, N.S., not significant, n = 3. [file 11658_2021_297_MOESM2_ESM.docx]

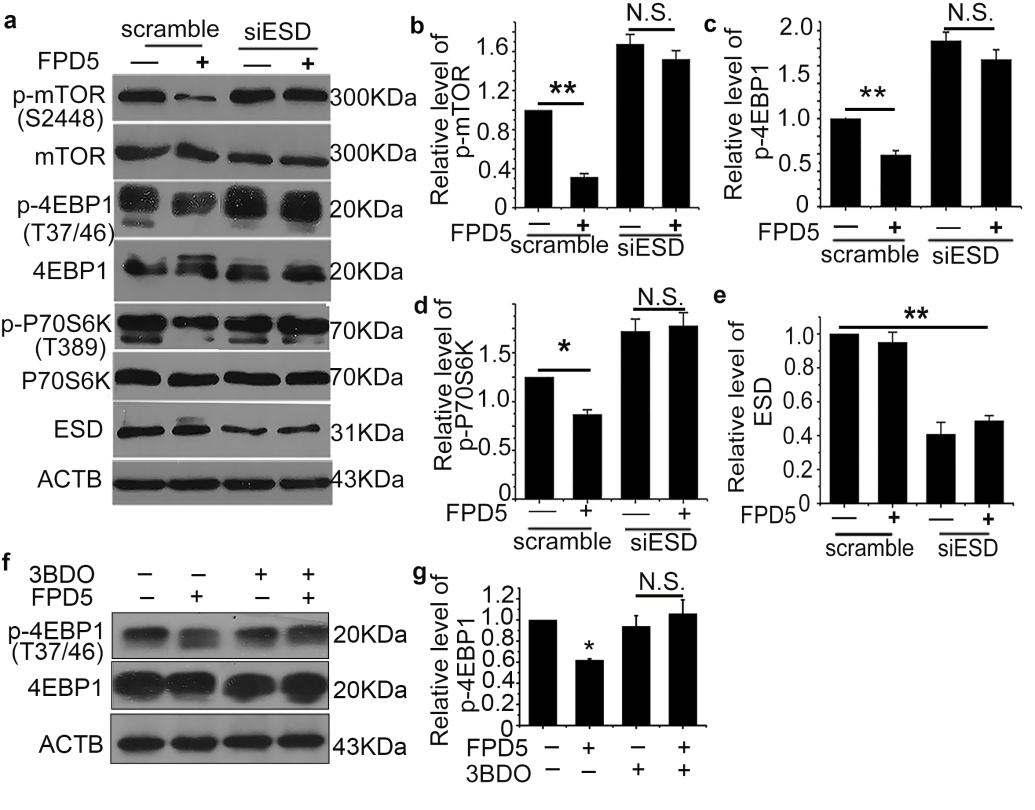


**Additional file 2: Fig. S2.** **FPD5 participated in regulation of mTORC1 activity via ESD.** (**a-e**) Western blot analysis of mTORC1, phosphorylated mTOR (p-mTOR, Ser2448), 4EBP1, phosphorylated 4EBP1 (p-4EBP1, Thr37/46), p70S6K and phosphorylated p70S6K (p-p70S6K, Thr389) level in A549 cell treated with FPD5 at 5 μM for 24 h after transfected with scramble siRNA or specific siRNA for ESD (siESD). (**f-g**) Western blot analysis of 4EBP1 and phosphorylated 4EBP1 (p-4EBP1, Thr37/46) level in A549 cell treated with FPD5 at 5 μM and 3BDO for 24 h. Data are mean ±SEM. *p < 0.05, **p < 0.01, N.S., not significant, n=3.
