# Supplementary material for: Esterase D stabilizes FKBP25 to suppress mTORC1
Source: Cell Mol Biol Lett. 2021 Dec 7;26:50. doi: 10.1186/s11658-021-00297-2 (PMC8903700; doi:10.1186/s11658-021-00297-2)
Supplement: Supplementary file 3 — Additional file 3: Figure S3. FKBP25 suppressed autophagy. (a–c) Western blot analysis of MAP1LC3BII and SQSTM1 level in A549 cell after transfected with scramble siRNA or specific siRNA for FKBP25 (siFKBP25). (d) Western blotting analysis of ESD and FKBP25 in HEK293T cells transfected with scrambled siRNA (scramble) or siRNA-FKBP25 (siFKBP25) for 24 h. Relative protein levels of FKBP25 and ESD is a ratio to ACTB (e–f). (g, h) Western blot analysis of MAP1LC3BII and SQSTM1 level in A549 cell treated with FPD5 at 0–5 μM for 24 h after transfected with myc-FKBP25. (i, j) HEK293T cell line was transfected with plasmids FKBP25-WT and the 1–90 aa FKBP25 for 24 h respectively. Then Western blot analyzed the levels of p-4EBP1 and 4EBP1. Data are mean ± SEM. *p < 0.05, **p < 0.01, ***p < 0.001, N.S., not significant, n = 3. [file 11658_2021_297_MOESM3_ESM.docx]

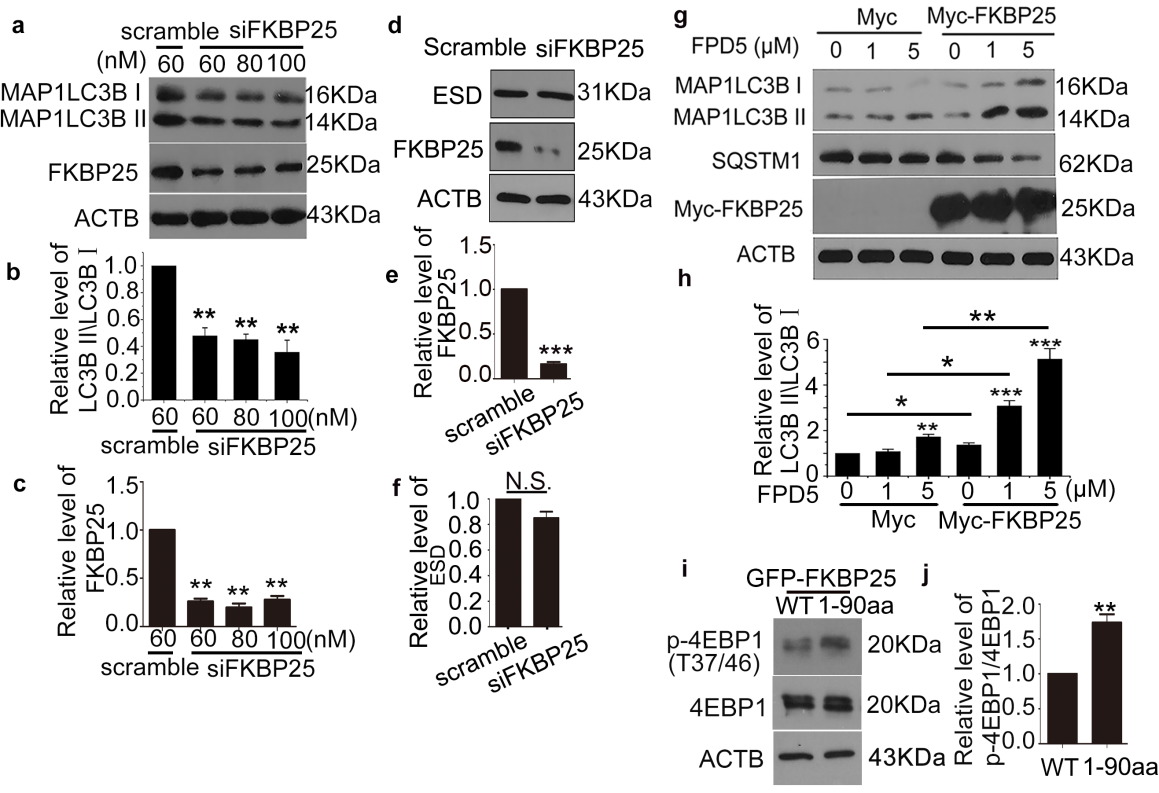


**Additional file 3: Fig. S3. FKBP25 suppressed autophagy.** (**a-c**) Western blot analysis of MAP1LC3BⅡ and SQSTM1 level in A549 cell after transfected with scramble siRNA or specific siRNA for FKBP25 (siFKBP25). (**d**) Western blotting analysis of ESD and FKBP25 in HEK293T cells transfected with scrambled siRNA (scramble) or siRNA-FKBP25 (siFKBP25) for 24 h. Relative protein levels of FKBP25 and ESD is a ratio to ACTB (**e-f**). (**g-h**) Western blot analysis of MAP1LC3BII and SQSTM1 level in A549 cell treated with FPD5 at 0-5 μM for 24 h after transfected with myc-FKBP25. (**i-j**) HEK293T cell line was transfected with plasmids FKBP25-WT and the 1-90aa FKBP25 for 24 h respectively. Then Western blot analyzed the levels of p-4EBP1 and 4EBP1. Data are mean ±SEM. *p < 0.05, **p < 0.01, ***p < 0.001, N.S., not significant, n=3.
