# Supplementary material for: Esterase D stabilizes FKBP25 to suppress mTORC1
Source: Cell Mol Biol Lett. 2021 Dec 7;26:50. doi: 10.1186/s11658-021-00297-2 (PMC8903700; doi:10.1186/s11658-021-00297-2)
Supplement: Supplementary file 4 — Additional file 4: Figure S4. ESD reduced the K48-linked polyubiquitination of FKBP25 after treatment with FPD5 at 5 μM for 24 h. [file 11658_2021_297_MOESM4_ESM.docx]

**
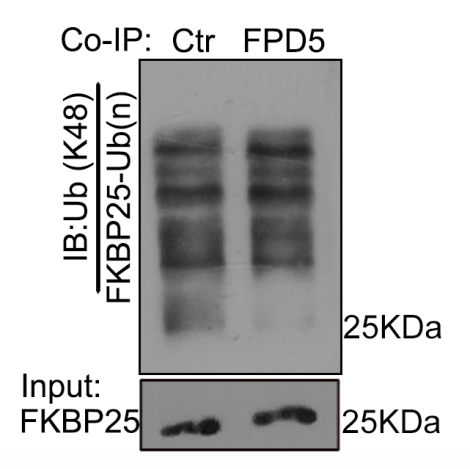
**

**Additional file 4: Fig. S4.** **ESD reduced the K48-linked polyubiquitination of FKBP25 after treatment with FPD5** **at 5 μM for 24 h.**
