# Supplementary material for: Esterase D stabilizes FKBP25 to suppress mTORC1
Source: Cell Mol Biol Lett. 2021 Dec 7;26:50. doi: 10.1186/s11658-021-00297-2 (PMC8903700; doi:10.1186/s11658-021-00297-2)
Supplement: Supplementary file 5 — Additional file 5: Figure S5. FPD5 did not induce apoptosis and necrosis. (a–c) FPD5 at 1–10 μM for 24 h decreased A549, H322 and HeLa cell viability. (d–f) Western blot analysis of cleavage PARP and Bax level in normal HUVEC cells treated with FPD5 at 0.1–10 μM for 24 h. (g) Lactate dehydrogenase (LDH) assay were performed in cancer cells with FPD5 at 10 μM for 24 h. (h, i) Biomicroscopy and quantification of angiogenesis on gelatin sponge with FPD5 adsorption. Scale bar: 1.5 mm. Data are mean ± SEM. N.S., not significant, n = 3. [file 11658_2021_297_MOESM5_ESM.docx]

**
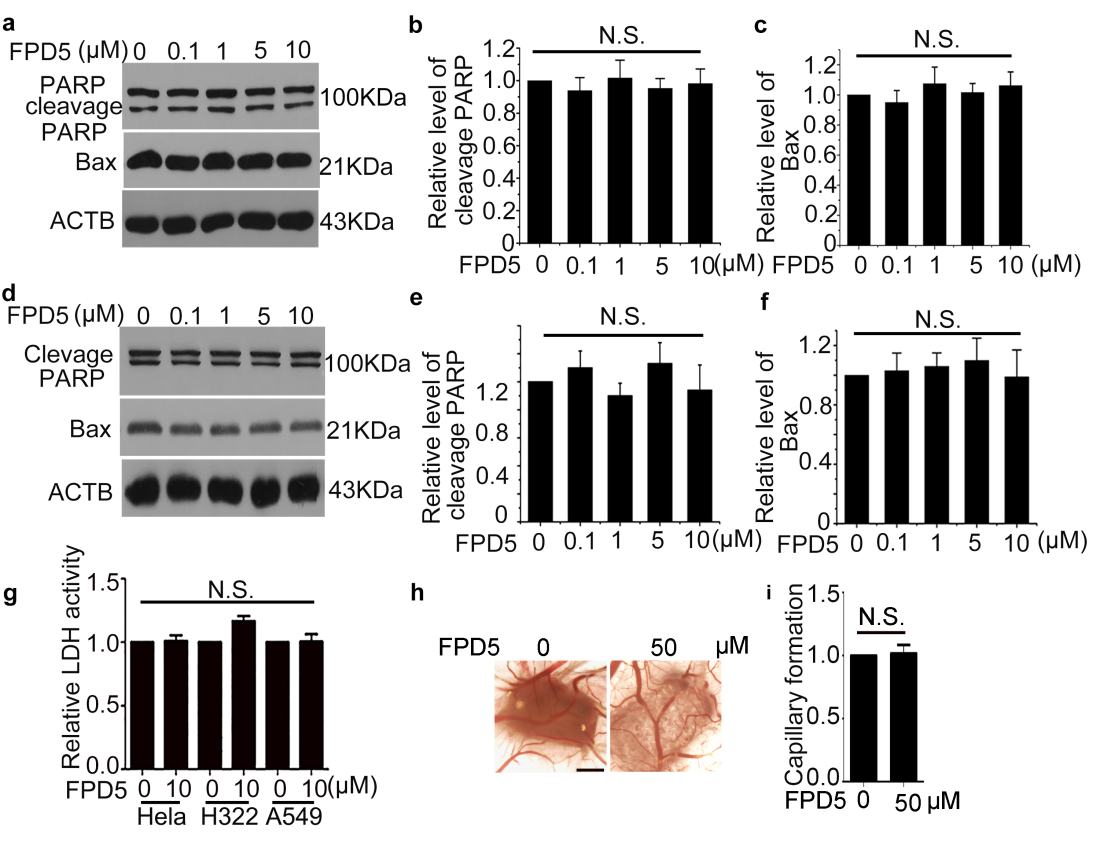
**

**Additional file 5: Fig. S5.** **FPD5 did not induce apoptosis and necrosis.** (**a-c**) FPD5 at 1-10 μM for 24 h decreased A549, **H322** and HeLa cell viability. (**d-f**) Western blot analysis of cleavage PARP and Bax level in normal HUVEC cells treated with FPD5 at 0.1-10 μM for 24 h. (**g**) Lactate dehydrogenase (LDH) assay were performed in cancer cells with FPD5 at 10 μM for 24 h. (**h-i**) Biomicroscopy and quantification of angiogenesis on gelatin sponge with FPD5 adsorption. Scale bar: 1.5 mm. Data are mean ± SEM. N.S., not significant, n=3.
